# Maternal depression and attachment: the evaluation of mother–child interactions during feeding practice

**DOI:** 10.3389/fpsyg.2015.01235

**Published:** 2015-08-24

**Authors:** Alessandra Santona, Angela Tagini, Diego Sarracino, Pietro De Carli, Cecilia S. Pace, Laura Parolin, Grazia Terrone

**Affiliations:** ^1^Department of Psychology, University of Milano-Bicocca, MilanItaly; ^2^Department of Educational Science, University of Genoa, GenoaItaly; ^3^Department of Humanities, Literature, Cultural Heritage, Education Sciences, University of Foggia, FoggiaItaly

**Keywords:** depression, attachment, mother–child relation, feeding

## Abstract

Internal working models (IWMs) of attachment can moderate the effect of maternal depression on mother–child interactions and child development. Clinical depression pre-dating birthgiving has been found to predict incoherent and less sensitive caregiving. Dysfunctional patterns observed, included interactive modes linked to feeding behaviors which may interfere with hunger–satiation, biological rhythms, and the establishment of children’s autonomy and individuation. Feeding interactions between depressed mothers and their children seem to be characterized by repetitive interactive failures: children refuse food through oppositional behavior or negativity. The aim of this study was to investigate parenting skills in the context of feeding in mothers with major depression from the point of view of attachment theory. This perspective emphasizes parents’ emotion, relational and affective history and personal resources. The sample consisted of 60 mother–child dyads. Mothers were divided into two groups: 30 with Major Depression and 30 without disorders. Children’s age ranged between 12 and 36 months The measures employed were the Adult Attachment Interview and the Scale for the Evaluation of Alimentary Interactions between Mothers and Children. Insecure attachment prevailed in mothers with major depression, with differences on the Subjective Experience and State of Mind Scales. Groups also differed in maternal sensitivity, degrees of interactive conflicts and negative affective states, all of which can hinder the development of adequate interactive patterns during feeding. The results suggest that IWMs can constitute an indicator for the evaluation of the relational quality of the dyad and that evaluations of dyadic interactions should be considered when programming interventions.

## Introduction

Attachment theory (Bowlby, 1973) provides a useful interpretative model for describing how experiences in early childhood can influence the development of caregiving skills. Bowlby (1973) postulated that caregivers’ Internal working models (IWMs), derived from the relationship with their own attachment figures during infancy and childhood, could directly influence their ability to respond sensitively to their children.

Maternal IWMs seem to be particularly relevant in pregnancy and early motherhood. Pregnancy may activate the women’s identification with past significant others, in particular with their own mothers (Innamorati et al., 2010). More specifically, it has been underlined that the presence of a potentially positive maternal representation may be crucial (Stern, 1995) for the implicit regulation of both the remembered and current relationship to one’s mother figure (Tambelli et al., 1995; Bifulco et al., 2002; Ammaniti et al., 2004).

Insecurity of attachment may also influence the way women subjectively experience their pregnancy, giving rise, for example, to ambivalent emotions toward the fetus and their future maternal role. This may be attributed to a reactivation of the future mothers’ representations related to their childhood experiences. These experiences may emphasize feelings of incompleteness and inadequacy, potentially contributing to negative emotions, and even to depressive states (Gerlsma and Luteijn, 2000; Bifulco et al., 2004; Cassidy et al., 2010; Hammen et al., 2012). These processes may be more relevant for young mothers, considering that a strong association between insecurity of attachment and internalizing problems, including depression, has been found in adolescents and young adults (Allen, 2008; Sarracino et al., 2011).

A number of studies conducted during the last two decades focused on the role of maternal attachment insecurity as a moderator variable on maternal depression and its effects on the psycho-emotional development of children (see, for example, McMahon et al., 2015). These studies suggest that mothers’ IWMs moderate the effect of maternal depression on their children’s development (Reis and Grenyer, 2004; Stansfeld et al., 2008a; Niolu et al., 2010). McHale (2007) found that depressive symptoms in one of the caregivers constitutes a risk factor for the quality of caregiving. More in general, studies have highlighted how having families in which a member suffers from mental disorders represents a risk factor for the individual, although in interaction with other vulnerability factors (Andersson et al., 2003; Heron et al., 2004; Austin et al., 2007; Faisal-Cury and Rossi Menezes, 2007; Lee et al., 2007).

Mothers’ psychopathology, and maternal depression in particular, may affect the development of the child directly or indirectly (Gross et al., 2008; Cho et al., 2015). Further, the chronicity and the severity of the disorder (Seifer and Dickstein, 2000; Grant et al., 2008) seem to have a greater impact than the diagnosis itself. In general, maternal depression is regarded as a “long-lasting vulnerability” (Karney and Bradbury, 1995), which may affect caregivers’ abilities to cope with difficulties inherent to assuming a parental role. It may for instance increase the likelihood of interpreting events as stressful and terrifying.

Attachment theory (Ainsworth et al., 1978) introduced the concept of sensitive responsiveness to evaluate a mother’s ability to intuitively identify and respond to the signals of her child with empathy. An updated version of this concept was proposed by Oppenheim and Koren-Karie (2009) and Quitmann et al. (2011), by referring to *insightfulness* as a specific caregiving skill that allows caregivers to take their children’s point of view. This skill implies being able to form dynamic representations of one’s child, within a relationship in which security and differentiation are facilitated. Depressed mothers, with specific deficits in *insightfulness*, may be confused by the emotional reactions of their children, and may be unable to distinguish between their own emotions and those of the children (Koren-Karie et al., 2002; Quitmann et al., 2011; Beebe and Lachmann, 2014). This distress may be pervasive and thus negatively influence their caregiving attitudes and behaviors (Agostini et al., 2014; De Campora et al., 2014).

Numerous studies highlighted that maternal depression, when associated with insecure attachment, can interfere with the quality of mother–child dyadic interactions (for a review, see Field, 2010). As suggested by several models and relevant empirical studies, in fact, the impact of maternal psychopathology on a child’s affective development is not unidirectional. Instead the effect should be considered as the result of interactions, occurring within dyadic systems (Herwig et al., 2004; Hoffman et al., 2006; Beebe and Lachmann, 2014; McCullough and Shaffer, 2014). Tronick (2005), for example, proposed a model in which the caregiver–child dyad is considered to be an affective communication system, in which mutual regulations take place. The aim of the system is to realize a “conscious dyadic state,” which is believed to influence the child’s representations as well as its emotional and social development. According to Tronick (2005), depressed mothers are unable to understand their children’s affective communications, and thus fail in attuning to these. Negative affects thus become pervasive within the dyad, stabilizing the negative affect within the child. The child will therefore interact negatively with the mother, and a mutual amplification of prolonged negative emotions will ensue. Depressed mothers thus tend to be less capable of communicating and sharing positive emotions, and more vulnerable to the distress of their infants (Goodman et al., 2011; Beebe and Lachmann, 2014). The depressed caregiver tends to discourage interactions with her child, and this may on the one hand not allow the child to integrate aspects of the relationship that are fundamental for personality-development; on the other hand, the caregiver may interfere with the child’s avoidant behavior, as it seems to confirm the mother’s sense of being unwanted. This, in turn, may reinforce the depressive condition by coloring it with feelings of aggression and rejection (Stansbury and Sigman, 2000; Cole et al., 2004).

The depressed mother’s behaviors can vary: some depressed mothers are intrusive and shows angry facial expressions, while others expresses sadness and withdrawal. The studies of Tronick and Weinberg (1997) assessed the different effects on the child of at least two patterns of interaction of depressed mothers: intrusiveness and rage, and sadness and withdrawal. Both modes of interaction interfere with the process of regulation and constitute a rupture in inter-subjectivity. Intrusive mothers tend to treat their children severely, address their children with angry tones of voice, and actively interfere with their activity. In contrast, withdrawn mothers tend to interact to lesser degrees, are emotionally flat, not reactive and do not support their children’s activities.

Tronick (2005) suggested that, when the experience of inter-subjectivity is distorted and marked by negative emotions, as in the case of the relationship of the child with a depressed mother, the child may incorporate elements of the mother’s negative emotional states. More generally, the affective states and behaviors of the children with non-depressed mothers are described as being more vital, responsive and assertive, when compared with those of children of depressed mothers. These interactions in turn facilitate feelings of efficacy and adequacy in the caregiver that encourage shared experiences of mutual satisfaction. In contrast, the depressed mothers frequently describe their children as being less vital and introverted. In particular, the children of intrusive mothers avoid their mothers’ gaze, rarely attend to objects, and rarely cry. On the contrary, the children of withdrawn mothers protest and tend to express their distress, suggesting that the withdrawn behavior has a particularly negative effect (Tambelli et al., 2010; Terrone and Santona, 2013). In the subsequent phases of development, the children of depressed mothers may show withdrawal, sadness and hostility, as well as externalizing problems such as aggression and anger (Weissman et al., 2006; Cho et al., 2015). The caregivers’ negative expectations related to their parental role and behavior are thus confirmed (Stansbury and Sigman, 2000; Cole et al., 2004; Simonelli et al., 2008; Goodman et al., 2011).

In particular, dysfunctional behavioral patterns have been found to characterize feeding interactions between depressed mothers and their children. Clinical studies have shown that children of depressed mothers are often unable to regulate their feeding rhythm, and tend to reject feeding (Chatoor et al., 2000; Stein et al., 2001). In these situations, the dyads fails to establish the essential shared rhythm during feeding, and the children do not learn to regulate their growing needs of autonomy and agency. The caregivers, in these situations, may be extremely controlling, and even scold and criticize the children. Children may thus fail to learn strategies by means of negotiation, and this creates a conflict between their need of autonomy and their mother’s rigidness (Chatoor et al., 2004; Haycraft and Blissett, 2008; Ammaniti et al., 2010).

### Aims and Hypotheses of the Study

In line with the above-mentioned models and empirical research, this study aimed to explore the dimensions correlated with parenting skills in a sample of depressed mothers.

More specifically, we explored the differences between depressed and non-depressed mothers regarding the mothers’ states of mind relative to attachment. Our hypothesis was that the clinical group would be more insecure on the Adult Attachment Interview (AAI) scales than the control group.

Second, we observed and coded mother–child interaction during feeding. Our hypothesis was that the dyads of the clinical group reported higher levels of problematic behavior, both for mothers and children.

A third aim was to investigate the maternal descriptions of their children. The hypothesis was that depressed mothers tend to have more negative representations of their children, describing them in terms of their lack of responsiveness and vitality.

## Materials and Methods

### Participants

Sixty mothers and their toddlers were recruited for the study. Thirty mothers, who satisfied the criteria for Major Depression of the DSM-5 (American Psychiatric Association, 2013) were recruited at the Psychiatric Unit of the University Hospital of “Tor Vergata” in Rome. The mothers were diagnosed by means of the DSM-5 (SCID-5). The depressed mothers were aged between 28 and 39 years (*M* = 31.5; SD = 5.6), with children aged between 12 and 36 months (*M* = 26; SD = 2.9). Thirty mothers were recruited in public nursery schools of Rome in order to constitute a non-clinical sample. The non-clinical participants were chosen in order to balance clinical mothers for gender and age of the children.

Gestation periods and children’s psychomotor development were within the norm in both groups. Most children had been breast-fed (clinical group = 74%; control group = 78%).

Participants tended to be married (clinical group = 90%; control group = 93%), had a Secondary school Diploma (clinical group = 74%; control group = 70%) or a University degree (clinical group = 13%; control group = 15%) and belonged to a middle socio-economic group (clinical group = 69%; control group = 73%; the SES was assessed in accordance with Hollingshead’s, unpublished manuscript criteria).

### Variables and Measures

#### Assessment of the Attachment Patterns of Mothers

The attachment patterns of the mothers were evaluated by means of the AAI (George et al., 1985), a semi-structured interview, that assesses and classifies an adult’s state of mind regarding attachment, by means of 20 questions. Participants are required to describe their relationships to caregivers mainly during childhood and support their assertions by recounting specific memories. Participants are also asked about events, activating the attachment system, such as separations from caregivers, any losses, or traumas. The interview also assesses the ability to reflect upon the effects of childhood experiences on development one’s current personality and caregiving. The AAI is audio-recorded and transcribed verbatim. The AAI transcript is evaluated according to the system developed by Main et al. (2002), which consists in providing scores from 1 to 9 on two groups of scales. Five scales refer to “probable past experiences” (Loving, Rejection, Neglecting, Role Reversal, and Pressure to Achieve), and 11 scales evaluate the “state of mind” with respect to attachment (Idealization, Lack of Memory, Anger, Derogation, Passivity, Transcript Coherence, Mental Coherence, Metacognitive Monitoring, Fear of Loss, Unresolved Loss, Unresolved Trauma). The transcripts are then assigned to one of three main categories: secure-autonomous (*free-autonomous*, F/A), Insecure/Distancing (*dismissing*, Ds), Insecure/Concerned (*enmeshed*, E). There are two additional categories, the Unresolved/Disorganized relative to loss or trauma (*unresolved*, U) and Cannot Classify (*cannot classify*, CC) for unorganized states of mind.

#### Assessment of Mother–Child Feeding Interactions

The Observational Scale for Mother–Child Feeding Interactions (SVIA) measures a vast spectrum of interactive behaviors and identifies normal and/or risky relational modes in the dyad, during feeding (Lucarelli et al., 2002). The coding system of the Scale is applied to video-recordings lasting 20 min, during the feeding of a child, aged 1–36 months. Since the SVIA is applied to children aged 1–36 months, developmental differences in behavior are considered through specific age-appropriate items. The studies conducted to assess the psychometric properties of the US and Italian version of the Scale found a good inter-rater reliability and a satisfactory construct and discriminant validity (Chatoor et al., 1997; Lucarelli et al., 2002).

The Italian version of the Scale is composed of 40 items. Each item is scored on a four-point Likert scale (never, a few times, often, very often). The items are grouped in four subscales: Affective State of Mother (difficulties of the caregiver in expressing positive emotions and the frequency and quality of negative affects); Interactive Conflict (presence and intensity of conflictual exchanges within the dyad); Food Rejection Behavior on behalf of the child (single characteristics of the feeding patterns of the child, e.g., rejection of food, poor food intake, and difficult regulation of states during the meal); Affective State of the Dyad (problems in the mother–child relationship).

#### Assessment of Child Emotional/Behavioral Functioning

The Child Behavior Check List (CBCL), 1½–5 (Achenbach and Rescorla, 2001) assesses behaviors and emotions of children in a number of areas of their functioning. The data is provided by the parents, who evaluate the statements on the CBCL 1½–5. The daily activities assessed by the 99 items include interests, attention, fears, playing, interactions with peers and adults, anxiety, physical problems, moods, aggression, affective responses and reactions to change. These evaluations lead to an assessment on the following scales: Internalizing, Externalizing, and Neither Internalizing Nor Externalizing scales. The Internalizing scale includes Emotionally Reactive, Anxious/Depressed, Withdrawn, Somatic Complaints; the Externalizing scale includes Attention Problems and Aggressive Behavior. The Neither Internalizing Nor Externalizing scale identifies problems that are not exclusively associated with other symptoms on the Internalizing or Externalizing scales. Every item is scored on a three-point Likert scale (0 = not true, 1 = partly true, 2 = very true). The behaviors identified refer to observations that occurred not more than 2 months previously to scoring.

### Procedure

All procedures followed were in accordance with the ethical standards of the Helsinki Declaration of 1975, as revised in 2000. Informed consent was obtained from all adult participants.

The AAI and the CBCL 1–5 were administered to all mothers. The administration and the coding of the AAI were carried out by psychologists, blind to the diagnoses of the mothers. Subsequently, the mother–child dyads were videotaped during mealtime for 20 min and they were coded using the SVIA. The videotapes were also assessed and coded by two independent researchers, blind to the diagnosis.

For our study, all the AAI’s and the SVIA’s were coded by certified coders, which are also author of this paper. For our study, all the AAIs were coded by a reliable coder. For inter-rater reliability, 30 interviews (50%) were also classified by another expert evaluator. Both coders were provided with AAI’s reliability and unaware of the other data collected. Inter-rater agreement was 82.5% (*k* = 0.62, *p* < 0.01) for four-way classifications (free-autonomous, dismissing, entangled, unresolved). For the SVIA, 30 video-recordings (50%) were coded. Inter-rater agreement was 90% (*k* = 0.85, *p* < 0.01) on the four subscales: Affective State of Mother, Interactive Conflict, Food Rejection Behavior on behalf of the child, Affective State of the Dyad.

### Data Analysis

The inferential analysis was carried out by means of permutation-based (i.e., non-parametric) univariate and multivariate tests (Pesarin, 2001). The approach was as follows: For each subscale, a univariate test was computed (10,000 random permutations). The MANOVA-like tests, which compare the measures (i.e., SVIA, CBCL, AAI and the subscales of AAI: Subjective experience, States of mind parents, and Overall states of mind) among the two groups, were obtained by means of non-parametric combinations of univariate tests (using the Fisher combining function), referring to the subscales of the measures themselves. The large quantity of tests performed required a correction of *p*-values for multiplicity. Furthermore, the analysis had a hierarchical structure for the overall, measures (i.e., SVIA, CBCL, and AAI) and subscales. The AAI had further levels: Subjective experience, States of mind, and Overall states of mind. The multiplicity correction was accomplished by the use of the min-p method (Westfall and Young, 1993) over all tests – i.e., both univariate and multivariate. To take into account the hierarchical nature of the analysis, the results will be discussed in a hierarchical order: if the overall adjusted-value was significant, the test for the three measures (i.e., SVIA, CBCL, and AAI) will be examined. Furthermore, the univariate tests for the subscales of each measure will be discussed only if the test of their associated measure was significant (e.g., the Loving-father scale is discussed as significant, only when the overall *p*-values of the Subjective experience scales on the AAI were significant after correction). The analysis continued hierarchically as long as all previous levels were significant after correction.

Note that the *p*-values related to measures (i.e., SVIA, CBCL, and AAI) were obtained by a combination of univariate tests on their subscales and not – for example – by comparing differences in frequencies of the clinical categories. The multivariate approach is usually more powerful than the latter and provides a more detailed understanding of the data.

To visually summarize the inferential results, we performed an explorative factor analysis on all (standardized) subscales, with principal components estimation method. Several considerations can be drawn from the biplot of Factor 1 vs. Factor 2 – accounting for 18 and 10% of the total variance, respectively.

The depressed (green dots) and control mothers (blue dots) are clearly separated in the biplot. This confirms results of the inferential analysis that shows a strong significance in the overall comparisons of the two groups. The subscales that are significant in the inferential analysis are highlighted with red arrows in the biplot. For each arrow, the direction indicates – roughly – the group with higher value; for example, the SVIA factors and the Rejecting.mt/ft are right oriented, indeed the depressed group has higher values in these scales.

All analysis were performed with R software (R Core Team, 2015) using library flip (Finos, 2014).

## Results

### Descriptive Analysis of Attachment Models

For descriptive purposes, we present the distributions of the attachment models in the experimental and control group. The unresolved classifications for trauma or loss (U) and the unclassified attachment (CC) were coded and collapsed into one U/CC category. Generally, a prevalence of insecure models was found in the experimental group. The frequency of the secure state of mind/autonomous in the control group (*N* = 18; 60%) was higher than in the depressed mother group (*N* = 10; 33%). The distancing state of mind (*N* = 10; 33%) was higher in the experimental group than in the control group (*N* = 5; 17%). The depressed mothers also had a higher incidence of the unresolved/unclassified attachment models (*N* = 5; 17%), which is typical of clinical samples, than the non-clinical group (*N* = 1; 3%).

### Statistical Analysis of the AAI Scales

The inferential analysis on the AAI scales highlighted significant differences between the experimental and control group, relative to the “Subjective experience” scale and the “Overall state of mind” scale, but not regarding the “Parents’ state of mind” scale (see “**Tables 1** and **2**”). Concerning the Subjective experience scales, we found significant differences on the Father Loving scales, with the control group having higher mean scores. The depressed group had higher mean scores on the rejection scales of mother and father and on the neglecting scale for mother. Significantly higher mean scores were found in the control group for the following Overall state of mind scales: i.e., metacognitive abilities and derogation of attachment. Depressed mothers also had significantly higher scores on the Lack of Memory Scale than the control group mothers.

**Table 1:** Mean and SD of Control and Depression groups for each measures.

		Controls		Depression	
		Mean	SD	Mean	SD
Scale for Mother–Child Feeding Interactions (SVIA)	SVIA.factor1	8.30	2.22	13.97	3.91
	SVIA.factor2	6.13	3.29	14.10	3.71
	SVIA.factor3	9.63	4.38	14.77	4.66
	SVIA.factor4	3.27	2.42	6.67	3.88
Child Behavior Check List (CBCL)	Internal	14.60	4.67	8.03	4.02
	External	14.10	7.14	12.03	6.46
Adult Attachment Interview (AAI): subjective.experience	Loving.ft	5.35	1.30	2.62	1.41
	Loving.mt	4.38	2.28	2.93	1.19
	Rejecting.ft	1.60	0.84	3.28	1.87
	Rejecting.mt	1.59	0.84	3.18	2.33
	Role.ft	1.63	0.93	1.58	1.18
	Role.mt	1.92	1.37	2.12	0.99
	Preas.achieve.ft	2.80	1.01	2.08	1.73
	Preas.achieve.mt	2.37	1.50	1.45	1.25
	Neglecting.ft	2.60	1.74	3.50	2.46
	Neglecting.mt	2.05	0.79	3.98	2.67
AAI: states.mind. parents	Idealizing.ft	2.40	0.77	2.63	1.89
	Idealizing.mt	2.48	0.55	3.33	1.96
	Anger.ft	2.15	0.90	2.20	0.52
	Anger.mt	1.98	0.55	2.53	0.87
	Derogation.ft	2.73	0.75	3.22	1.59
	Derogation.mt	2.72	0.75	2.73	1.10
AAI: overall.states. mind	Global.Derogation	2.22	0.73	1.41	0.75
	Lack.memory	2.00	0.67	3.12	1.53
	Meta.cognition	3.12	1.22	1.77	0.75
	Passivity	2.85	1.15	2.73	1.36
	Fear.Loss	1.29	0.66	1.55	1.01
	U.abuse	2.47	0.81	1.96	1.50
	U.loss	1.68	0.47	2.28	1.54
	Coh.Trans	4.30	1.51	3.95	1.05
	Coh.Mind	4.68	1.09	3.97	1.17

**Table 2:** Comparison of the Control and Depression groups: inferential results.

	Statistical test	Statistics	*p*-value	Adjustment:min P	Significance
Overall	Fisher	140.9281	0.0001	0.0025	**
SVIA	Fisher	36.1482	0.0001	0.0025	**
svia.factor1	*t*	6.9089	0.0001	0.0025	**
svia.factor2	*t*	8.8053	0.0001	0.0025	**
svia.factor3	*t*	4.4065	0.0001	0.0025	**
svia.factor4	*t*	4.0720	0.0002	0.0048	**
CBCL	Fisher	9.2103	0.0001	0.0025	**
internal	*t*	-1500.5228	0.0001	0.0025	**
external	*t*	-472.2458	0.2531	0.9313	
AAI	Fisher	94.1956	0.0001	0.0025	**
*subjective experience*	Fisher	49.4500	0.0001	0.0025	**
loving.ft	*t*	-7.8153	0.0001	0.0025	**
loving.mt	*t*	-3.0849	0.0032	0.0570	
rejecting.ft	*t*	4.4853	0.0002	0.0048	**
rejecting.mt	*t*	3.5195	0.0003	0.0066	**
role.ft	*t*	-0.1829	0.9054	0.9978	
role.mt	*t*	0.6480	0.5306	0.9917	
preas.achieve.ft	*t*	-1.9598	0.0522	0.5241	
preas.achieve.mt	*t*	-2.5763	0.0131	0.1976	
neglecting.ft	*t*	1.6411	0.1060	0.7655	
neglecting.mt	*t*	3.7835	0.0005	0.0109	*
*states.mind.parents*	Fisher	11.7444	0.0241	0.3078	
idealizing.ft	*t*	0.6175	0.5585	0.9917	
idealizing.mt	*t*	2.2896	0.0261	0.3214	
anger.ft	*t*	0.2633	0.8602	0.9978	
anger.mt	*t*	2.9275	0.0046	0.0785	
derogation.ft	*t*	1.5052	0.1391	0.8126	
derogation.mt	*t*	0.0687	0.9888	0.9978	
*overall.states.mind*	Fisher	33.0011	0.0001	0.0025	**
global.derogation	*t*	-6.6789	0.0015	0.0288	*
lack.memory	*t*	3.2099	0.0005	0.0109	*
meta.cognition	*t*	-5.1532	0.0001	0.0025	**
passivity	*t*	-0.3589	0.7605	0.9967	
Fear.loss	*t*	0.6225	0.4832	0.9917	
u.abuse	*t*	1.7404	0.2223	0.9204	
u.loss	*t*	1.5770	0.1306	0.8126	
coh.trans	*t*	-1.0403	0.3045	0.9500	
coh.mind	*t*	-2.4539	0.0191	0.2601	

Used statistical test, computed test statistics, (unadjusted) *p*-values and adjusted *p*-values are reported for each subscale and each scale. *p < 0.05, **p < 0.01.

### Mother–Child Feeding Interaction

The assessment of the mother–child food interaction, by means of the SVIA, evidenced that the dyads of the clinical group reported higher points than the control group in the following scales: Food Rejection Behaviors of the Child (SVIA factor1), Interactional Conflict (SVIA factor2), Affective States of the Mother (SVIA factor3), and Affective State of the Dyad (SVIA factor4). (See “**Tables 1** and **2**”).

### Emotional–Behavioral Problems in the Child

**FIGURE 1:**
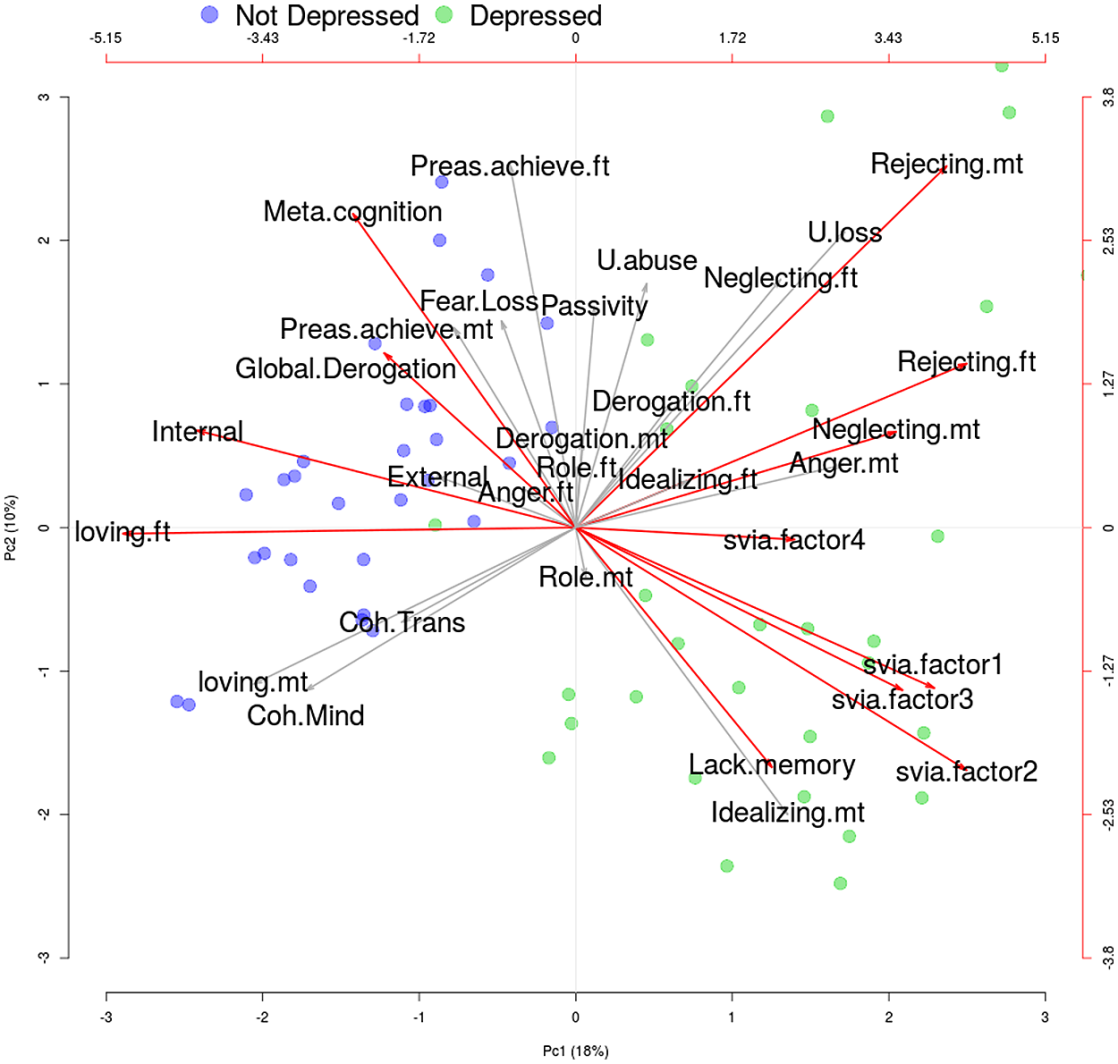
**Biplot of the factorial analysis (two factors).** Subscales which significantly differs between groups after correction for multiplicity – as reported in **Table 1** – are highlighted with a red arrow. Not Depressed and Depression groups are marked in blue and green, respectively. More details are given in the text.

Higher mean scores on both the Internalization and Externalization scales (see “**Tables 1** and **2**”) were found in the depressed group, when mothers evaluated their children’s adaptation and daily functions. Finally, “**Figure 1**” indicates the results of the principal component analysis by means of a biplot. The biplot visualizes the inferential results already discussed in detail. The two groups (depressed in green, controls in blue) are almost completely separated on the plain of the first two components (i.e., a very significant overall *p*-value). The scales with significant differences among groups are represented by red arrows. The depression group had higher values (i.e., red arrows on the right) on the following factors: *Rejecting.ft, Rejecting.mt, Neglecting.mt, Lack.memory, Svia.factor1, Svia.factor2*, *Svia.factor3*, *Svia.factor4*. On the contrary (red arrows on the left) they had lower values of *Neglecting.mt, Internal, Loving.ft, Global.Derogation, Meta.cognition*.

## Conclusion

The main aim of our study was to analyze the attachment models of depressed mothers in order to understand the role of insecure models in defining parental competence.

The results confirmed that the *security* variable was a principal factor in differentiating the experimental and control group. Insecure states of mind relative to attachment were, in fact, higher in the group of depressed mothers. The analysis of the scales of the AAI identified major areas of vulnerability, which are connected to peculiar development paths in the depressed mothers. These were characterized by specific representational structures of attachment, formed during the emotional–relational interactions with their own caregivers (Steele and Steele, 2008).

In particular, the scales of AAI suggested that the depressed mothers of the experimental group perceived their fathers as having provided inadequate affective experiences, insufficient love, and in general, as having been a caregiver who was not emotionally supportive. Indeed, the Affective deprivation during infancy is a risk factor for the development of both insecure attachment and depressive disorders (Bifulco et al., 2002). Retrospective studies suggest that experiencing affective deprivation, and perceiving and remembering inadequate paternal care, may compromise the relationship between fathers and daughters (Stansfeld et al., 2008b). This evidence suggests that both parents affect the IWMs of their children, albeit in different ways (Baldoni et al., 2009; Cummings et al., 2013). These participants, also represented both parents as having been rejecting, their mothers as neglecting, and thus less sensitive and attentive to their childhood needs. In our study, metacognitive knowledge was more deficient in depressed mothers. These mothers tended to limit the influence of their childhood relationships to caregivers on their current thoughts, emotions and personality organization. Attachment theory (Main et al., 1991) postulates, that this process occurs by means of the deactivation of the attachment system. These results suggest a number of considerations relative to maternal competence in depressed mothers. Representational models of attachment to one’s own parents are thought to regulate the ability of parents to understand affective states in their children, as well as their responses to the children’s signals. Attachment styles characterized by contradictions, distortions or negative emotions could thus interfere with maternal competence, in particular, with the process of recognizing and attuning to their children’s needs (Fonagy and Target, 1997; Barone, 2007).

An unexpected result of the study was the significantly higher scores on the derogation of attachment scale found in the control group. Further research is needed to clarify this finding.

### Emotional–Behavioral Problems in the Child

The other variables considered in our research were the emotional–behavioral problems of the children, as evaluated by their mothers. The results revealed that the depressed mothers reported a higher incidence of emotional–behavioral problems in their children, in particular regarding internalizing problems. This evidence is in line with previous studies, showing that parenting characterized by “affectionless control” (typical of depressed mothers) seems to contribute to the development of internalizing problems in the child (Wardle et al., 2001; Cicchetti and Toth, 2009; Goodman et al., 2011). These parents have been found to be less warm, less involved and less attentive to caring for their child, and even to openly express rejection. This parenting style may influence the emotional–behavioral styles of the children, affecting their ability to modulate sensorial input, to maintain calm and positive affective states and ultimately to self-regulate emotions and behaviors.

The evidence of the children’s internalizing behaviors has to be placed in the context of significant relationships. This perspective, considers any kind of relational dysfunction as a predictive factor that can limit or distort the emotional–behavioral and social experiences of the child, putting at risk his adaptive potential in an everyday life context. In fact, depressed mothers tend to describe their children as relationally “difficult.” This finding is particularly interesting since it suggests the role of the child as an active and competent partner, able to influence the relationship with the mother, and as being inevitably influenced, in a complex interactive system characterized by reciprocity and mutual regulation (Chatoor et al., 2000; Goodman et al., 2011; Wynter et al., 2014).

### Evaluation of the Mother–Child Feeding Interactive Patterns

To better understand the mutual influences that some factors may have within the dyad, this study also focused on the bidirectional interaction during an episode of feeding. The relational modes in the depressed mothers–children group revealed several dysfunctional interactions – namely, interactive conflictual behaviors, controlling behaviors of mothers, repeated communication failures, and negative involvement of couples in the feeding pattern of their children. We can hypothesize that these difficulties hinder the establishment of a stable biological feeding rhythm and as a consequence, the processes of autonomy and individualization (Chatoor and Ammaniti, 2007; Ammaniti et al., 2010). This data is consistent with the specific “intrusive” pattern described by Tronick and Weinberg (1997).

Specifically, these mothers showed a deficit in their attunement and a negative emotional involvement, characterized by emotional withdrawal, sadness, and anger. The feeding exchanges between the depressed mothers and their children was characterized by repetitive interactive failures, in which the child manifested oppositional behaviors such as the rejection of food (Stein et al., 2001; Lucarelli et al., 2003; Chatoor, 2012).

The depressed mother–child dyads have been found to have difficulties in expressing positive emotions, and in reciprocally interpreting signals (Tronick, 2005; Beebe and Lachmann, 2014). The dyadic interactions can become intensely conflictual and asynchronous (Radesky et al., 2013). The lack of attunement can generate defensive controlling strategies in depressed mothers, who have difficulties in modulating and negotiating the conflictual interactions with their children, thus facilitating the rejection of food (Chatoor et al., 2000; Stein et al., 2001; Ammaniti et al., 2010).

Mothers who show an excessive psychological control of the child seem to deny or not recognize the psychological autonomy and individuality of their children (Barber and Harmon, 2002; Kerig, 2003). Control becomes an educational strategy used by the parent to persuade the child to obtain certain results. Mothers can be intrusive, controlling, and overprotective (Pomerantz and Eaton, 2001; Grolnick et al., 2002), thus inhibiting their children’s behavior and encouraging dependence. Parenting modalities can also be critical or openly rejecting, and the parent may control the feeding procedure without taking into account the child’s signals, or may seem to worry excessively about the “mess” the child makes during the meal (Mills et al., 2007). The adoption of controlling behaviors on behalf of mothers may also limit their ability to be supportive, by allowing them to explore the environment and to make autonomous decisions as to when to start eating for example. This kind of support has repeatedly been linked to the development of autonomy in the child (Grolnick et al., 2002; Campbell et al., 2007).

Our study is also consistent with previous literature on the link between controlling and intrusive parenting and vulnerability to internalizing problems (Barber, 1996; Barber and Harmon, 2002; Grolnick, 2003). During feeding, in fact, the children of depressed mothers in our study tended to respond to the control and to the overprotection of their mothers with rejecting behaviors (refusing to open their mouth, crying when the food was presented, moving the food away or throwing food), withdrawn behaviors (open discomfort, falling asleep, and stopping to eat), or avoiding behaviors (avoiding eye-contact, stiffening when touched; Chatoor et al., 2000; Bryant-Waugh et al., 2010; Kerzner et al., 2015). In summary, the pattern that prevailed in our clinical group was that of the intrusive mother and withdrawn child, similar to the ‘chase and dodge’ pattern, described by Beebe and Lachmann (2014). It is possible that, in a larger sample, other dysfunctional patterns between mother and child may occur.

In conclusion, it is important to point out that in our data, mothers with major depression were also less flexible in adapting to the changes linked to parenthood. These major difficulties of adaptation to their new role could also lead to fractures in the interactive process, limiting competent interactions between mother and child, in which the child can self-regulate (Galloway et al., 2005; Kreipe and Palomaki, 2012). At the same time, these children were exposed to less warmth and more hostility. The mothers’ psychopathology, in fact, may determine chronic depressive moods and thus expose the child to prolonged inadequate interactive modalities (Rothschild and Zimmerman, 2002).

## Limits of the Study and Future Developments

The present study presents some limits. First of all, the size of our sample was not sufficient to draw conclusions regarding the complex interactions between the variables examined in our study. Secondarily, more recent studies and clinical practice strongly suggest considering the roles of fathers in family dynamics. Our study limited itself to mother–child interactions in order to be able to examine numerous variables within these dyads more extensively. Future research needs to integrate the father–child dyad, also in attachment terms. The assessment of the emotional-adaptive function of the children by means of a self-report questionnaire completed by mothers, also constitutes a limit. Although a part of the literature has insisted on the necessity to use objective instruments, or assessments administered by expert clinicians, it is also true that recent studies (for example, Bush et al., 2008) have suggested that mothers’ points of view – including depressed and attachment-disorganized mothers –, provide useful information as to their emotions and the emotions that prevail within the dyads. Nevertheless, future studies should integrate observational methods to a greater extent.

Finally, our clinical observation supported the two patterns of interaction of depressed mothers – intrusiveness and rage, and sadness and withdrawal – identified by Tronick and Weinberg (1997). However, we did not include these two maternal patterns in our data analysis. In the future developments of the study we are planning a specific comparison of these two kinds of maternal patterns (and a comparison of these two patterns vs. a control group of no-depressed mothers).

## Conflict of Interest Statement

The authors declare that the research was conducted in the absence of any commercial or financial relationships that could be construed as a potential conflict of interest.
